# Differences in the Prevalence of and Factors Associated with Frailty in Five Japanese Residential Areas

**DOI:** 10.3390/ijerph16203974

**Published:** 2019-10-18

**Authors:** Takumi Abe, Akihiko Kitamura, Satoshi Seino, Yuri Yokoyama, Hidenori Amano, Yu Taniguchi, Mariko Nishi, Miki Narita, Tomoko Ikeuchi, Yui Tomine, Yoshinori Fujiwara, Shoji Shinkai

**Affiliations:** 1 Research Team for Social Participation and Community Health, Tokyo Metropolitan Institute of Gerontology, Tokyo 173-0015, Japan; 2 Japan Society for the Promotion of Science, Tokyo 102-0083, Japan; 3 Center for Health and Environmental Risk Research, National Institute for Environmental Studies, Tsukuba 305-0053, Japan; 4 Tokyo Metropolitan Institute of Gerontology, Tokyo 173-0015, Japan

**Keywords:** area difference, community, frail, living area, location

## Abstract

This study aimed to examine area differences in the prevalence of and factors associated with frailty. This cross-sectional study included metropolitan (eastern and western areas), suburban (districts A and B), and rural areas of Japan (*n* = 9182, woman 50.9%). Frailty was defined by using a standardized questionnaire comprising three subcategories (fall, nutritional status, and social activities). The prevalence of frailty in the five areas was 14.2% to 30.6% for men and 11.5% to 21.4% for women. The areas with a high frailty prevalence had a significantly lower nutritional status or social activity, or both. Compared to the western metropolitan area, among men, the multivariable-adjusted prevalence ratio (APR) of frailty was significantly higher in the eastern metropolitan area and lower in suburban district A, and among women, the eastern metropolitan and rural areas had significantly higher APRs. Area-stratified multiple Poisson regression analysis showed that age, bone and joint disease, and a subjective economic status were associated with frailty in most areas and that some factors were area-specific, i.e., living alone (for men living in metropolitan areas) and underweight (for women living in suburban areas). The frailty prevalence differed by area, even after multivariable adjustment. Area-specific characteristics and factors associated with frailty may result in area differences.

## 1. Introduction

Many countries aim to increase the life expectancy, and, in the future, the extension of a healthy lifespan will be a priority [1]. One target for extending a healthy lifespan is frailty—a state of increased vulnerability that can lead to adverse health outcomes, such as disability, hospitalization, and death [2]. A meta-analysis using different definitions of frailty (i.e., phenotype, deficit accumulation, and multidomain models) found that frailty was a significant predictor of adverse health outcomes, regardless of definition [3]. Therefore, frailty is an important public health issue [4].

Previous multinational studies have reported a wide range in the frailty prevalence, which suggests that it differs by area [5,6,7]. Two national surveys reported an urban/rural difference [8,9]. Data from nationally representative samples of Chinese people 60 years or older showed that the frailty prevalence was higher in rural areas than in urban areas (approximately 1.1 times for pre-frailty and 1.5 times for frailty) [8]. A national survey in Taiwan reported that the frailty prevalence was significantly higher among those living in a rural area than among those living in an urban area [9]. In contrast, a Chinese study showed that the frailty prevalence was significantly lower in a rural area than in an urban area of Beijing [10]. Another study, in Yogyakarta, Indonesia, found no difference in frailty status between urban and rural areas [11].

These area differences in frailty prevalence require further investigation because, although these previous studies investigated urban/rural differences in the frailty prevalence [8,9,10,11], the analyses were unadjusted or only adjusted for age. This indicates that the urban/rural differences in frailty may be explained solely by sociodemographic factors of the sample. Therefore, urban/rural differences in frailty among older adults should be confirmed in a multivariable-adjusted model. Second, if there are area differences in frailty prevalence in the model, the relevant factors should be identified. This would contribute to a reduction of the prevalence of frailty.

This study examined area differences in frailty prevalence in multivariable-adjusted models and attempted to identify factors associated with frailty in those areas.

## 2. Methods

### 2.1. Study Design and Participants

The present cross-sectional study used data collected from three Japanese areas: Ota city, Tokyo (population: 712,057 in 2016); Hatoyama town, Saitama Prefecture (population: 14,000 in 2018); and Kusatsu town, Gunma Prefecture (population: 6595 in 2017) [12]. In accordance with the classification of the Japanese population census [13], this study defined Ota city as a metropolitan area, Hatoyama town as a suburban area, and Kusatsu town as a rural area.

The present data for Ota City are baseline observational data from a community-wide intervention trial. The details of that study have been reported elsewhere [14]. Briefly, a baseline survey was conducted with an age- and sex-stratified random sampling design, in 2016, and self-reported questionnaires were distributed to 15,500 (intervention districts, *n* = 8000; control districts, *n* = 7500) older adults without long-term care insurance (LTCI) certification (i.e., no disability [15]). The current study used data from 6009 individuals, which were obtained from the intervention districts. After excluding missing data (*n* = 1224), the present study divided the intervention districts into western (two districts, *n* = 2436 out of 4000) and eastern (one district, *n* = 2349 out of 4000) metropolitan areas, because the western region included a high-income residential area. Therefore, income level and population characteristics differ between these areas. The data for Hatoyama town were gathered from completed surveys distributed in 2018. In the survey, self-reported questionnaires were distributed to older adults without LTCI certification living in Hatoyama town (*n* = 5150); 3335 consented to the survey. After excluding missing data (*n* = 514), we divided Hatoyama town into districts A (commuter town, *n* = 1950) and B (other areas, *n* = 871), because it was assumed that these districts had different income levels (district A has a high-income level) and characteristics (See Table 1 and Table 2). The data for Kusatsu town were obtained from the Kusatsu Longitudinal Study on Aging and Health, which has been described elsewhere [16,17,18]. In short, the study includes data from both health check-ups and complete surveys. If participants attended the health check-up and completed a questionnaire that doubled as the complete survey, in July 2017, the current study used those data. The complete survey was conducted in November 2017, and the survey data were used if participants did not attend the health check-up. Self-reported questionnaires were distributed at the health check-up and in the complete survey, and data were obtained from 2071 participants (1290 and 781 from the health check-up and complete survey, respectively). After excluding persons with LTCI certification or missing data (*n* = 495), we ultimately collected data from 1576 residents of Kusatsu town. Therefore, in total, data from 4504 men and 4678 women were analyzed (Figure S1). Each study was approved by the Ethics Committee of the Tokyo Metropolitan Institute of Gerontology (ref. 2016-8 for Ota city; ref. 2017-K42 for Hatoyama town, ref. 2015-jin23 for Kusatsu town).

### 2.2. Frailty Assessment

The Kaigo-Yobo Checklist (KYCL) was used to assess frailty in all five areas [16,19]. The KYCL comprises 15 yes/no questions and has three subcategories: falling (six questions), nutritional status (four questions), and social activities (five questions) (See Supplementary Table S1) [16]. A previous study confirmed a significant correlation between the KYCL and Frailty Index [20], and these two measures have almost the same predictive ability for incident disability and/or mortality [21]. A higher KYCL score indicates a worse status; the cut-off for frailty was 3/4.

### 2.3. Associated Factors

In this study, we analyzed age, underweight, overweight, medical history (hypertension, heart disease, stroke, diabetes, cancer, and bone and joint diseases), living arrangement (living alone), and subjective economic status as factors potentially associated with frailty. The body mass index (BMI (kg/m^2^)) was calculated from height and weight, and underweight (<18.5 BMI) and overweight (≥27.5 BMI) were defined by using criteria for Asian populations [22]. A subjective economic status was assessed with a five-point scale and dichotomized as poor (very poor or poor) or not poor (very good, good, and normal). The questionnaires distributed to all five areas included the same questions and options to obtain the information.

### 2.4. Statistical Analysis

All analyses were stratified by sex, because previous systematic reviews have reported sex differences in frailty [23,24], and were performed with SPSS version 23.0 (IBM, Armonk, NY, USA). A *p* value of <0.05 was considered to indicate statistical significance.

When comparing characteristics, we used the independent t-test for continuous variables and chi-square test for categorical variables. An analysis of covariance (ANCOVA) adjusted for factors associated with frailty was used to compare scores for KYCL subcategories. The independent t-test, chi-square test, and ANCOVA were repeated four times because the analyses compared the western metropolitan area with each of the other areas. Therefore, the *p* value for those analyses was adjusted with Bonferroni correction (a *p* value of <0.0125 was considered to indicate statistical significance).

Multiple Poisson regression analysis was performed to calculate adjusted prevalence ratios (APRs) for frailty and 95% confidence intervals (CIs). The five areas (with the western metropolitan area as the reference) and factors associated with frailty were included as independent variables simultaneously. Additionally, area-stratified multiple Poisson regression analysis was used to examine area-specific factors associated with frailty.

## 3. Results

### 3.1. Descriptive Data

In the five areas, the frailty prevalence ranged from 14.2% to 30.6% for men and from 11.5% to 21.4% for women (Table 1 and Table 2, respectively). With men in the western metropolitan area as the reference, those in the eastern metropolitan area had the highest frailty prevalence and were more likely to be living alone and to have a poor subjective economic status. In suburban district A, which had the lowest frailty prevalence, men were younger and less likely to have hypertension, heart disease, cancer, bone and joint disease, and a poor subjective economic status.

The trend was similar in women. Those in the eastern metropolitan area had the highest frailty prevalence; were less likely to be underweight; and were more likely to be overweight and have hypertension, diabetes, bone and joint disease, and a poor subjective economic status. Women in suburban district A had the lowest prevalence of frailty and were younger. Additionally, they were less likely to be underweight; to have hypertension, heart disease, and bone and joint disease; and to be living alone, and more likely to have a poor subjective economic status.

### 3.2. Scores for KYCL Subcategories

Table 3 shows the results of the comparison of KYCL subcategories. Among men, fall component scores were better for suburban district A (*p* = 0.011), the eastern metropolitan and rural areas had significantly worse scores for the nutritional status (*p* < 0.001), and the social activities component scores were better for suburban districts A and B and for the rural area (*p* < 0.001).

Among women, there was no significant difference in fall component scores between the western metropolitan area and any other area. As was the case for men, the eastern metropolitan (*p* < 0.001) and rural areas (*p* = 0.004) had significantly worse scores for the nutritional status. The social activities component scores were worse for the eastern metropolitan area (*p* = 0.004) and better for suburban districts A (*p* < 0.001) and B (*p* = 0.004).

### 3.3. The Association Between Area and Frailty

Table 4 shows the age- and multivariable-adjusted APRs for men and women. Among men, the APR for frailty was significantly higher for the eastern metropolitan area (APR 1.27; 95% CI 1.11–1.46) and lower for suburban districts A (APR 0.73; 95% CI 0.61–0.88) and B (APR 0.79; 95% CI 0.63–1.00) in the multivariable-adjusted model.

Among women, the eastern metropolitan area (APR 1.31; 95% CI 1.12–1.55) and rural area (APR 1.38; 95% CI 1.14–1.65) had significantly higher APRs for frailty in the multivariable-adjusted model.

### 3.4. Factors Associated With Frailty in Each Area

When the present data were combined, APRs in both sexes were significantly higher for all associated factors except overweight, hypertension, and living alone in women (see Supplementary Table S2). The APR for living alone was higher in men (APR 1.30; 95% CI 1.13–1.49), but lower in women (APR 0.84; 95% CI 0.72–0.98).

Among men, multiple factors were associated with frailty in the five areas. Overall, bone and joint disease and a poor subjective economic status were significantly associated with frailty in all five areas. The APR for underweight was significantly higher in all areas except the western metropolitan area. Overweight and living alone were only significantly associated with frailty in the two metropolitan areas.

Among women, age was significantly associated with frailty in all five areas, and bone and joint disease was significantly associated with frailty in all areas except the eastern metropolitan area. There was only a significant association between underweight and frailty in the two suburban areas. Stroke and diabetes were only associated with frailty in the suburban and rural areas.

## 4. Discussion

This study included metropolitan, suburban, and rural areas of Japan in an analysis of area differences in frailty in older adults. As with previous studies that showed area differences in the prevalence of frailty in unadjusted and age-adjusted models [8,9,10,11], we noted significant area differences in the frailty prevalence, even in the multivariable-adjusted models.

The frailty prevalence was highest in the eastern metropolitan area in both sexes, which was expected because a previous study reported that this area was less healthy than the western metropolitan area—the reference group in this study [14]. Although the previous analysis was unadjusted [14], the present multivariable-adjusted model showed the same trend. Among women, the prevalence of frailty was high in the rural area. Nutritional status is known to be associated with frailty [25] and was worse in the eastern metropolitan and rural areas in both sexes. Improvement of the nutritional status could prove to be an important strategy to prevent frailty in those areas. In particular, malnutrition tends to be more common in rural areas [26]. Therefore, this implication may apply to other rural areas.

Among men, the prevalence of frailty was lower in suburban districts A and B. Social activities component scores were better in those districts and in the rural area than in the western metropolitan area. Social function, including social activity, was associated with location. A large-scale Japanese study reported that older men and women living in metropolitan areas were less likely to interact with a friend and participate in group activities than those in urban and rural areas [27], indicating that the metropolitan area had a lower level of social activities. Our results support these previous findings. Social function is an aspect of frailty (i.e., social frailty) [28] and is bidirectionally associated with physical and cognitive function [29]. Therefore, the higher level of social activities is attributable to the lower prevalence of frailty in suburban districts A and B. In addition, the higher level of social activities in men in the rural area likely compensated for their worse scores in the other KYCL components (i.e., fall and nutritional status); hence, the APR for frailty was not significantly higher for men in the rural area. This suggests that promoting social activities in the whole community may be an effective strategy for reducing the prevalence of frailty in the area.

In most areas, a subjective economic status and bone and joint disease were significantly associated with frailty in both sexes. These factors were previously reported to be associated with frailty [23,30,31]. In the present study, underweight (BMI < 18.5), which is sometimes included as a component of frailty [29,30], was associated with frailty in most areas, especially in men. The association between living alone and frailty was unfavorable for men, but favorable for women. The impact of living alone on adverse health outcomes is confounded by social function [32]. The social activity level is higher in women than in men, as our results show, which might explain the present sex difference. Additionally, our results suggest that some area-specific factors are associated with frailty, such as underweight and living alone (among men living in the metropolitan areas) and underweight (among women living in the suburban areas). Although it is difficult to identify the reasons why area-specific factors were found, other factors that are not included in this study (e.g., food intake, physical activity, and depression) are likely associated with the results. Two previous studies reported factors relevant to frailty in urban and rural areas [9,10], but the effects of medical history were unclear. Interestingly, the details of the participants’ medical history were not consistent between areas in the present study. Indeed, the effects of medical history on frailty were inconsistent in previous studies [33,34]. A prospective Japanese cohort study reported that medical history was not a significant predictor of incident frailty^33^, while another cohort study found that some diseases, such as diabetes and cancer, significantly increased the risk of frailty [34]. The present and these previous studies did not consider disease severity. Individuals with a severe disease might have been excluded from this study because they were more likely to need long-term care—one of the exclusion criteria in the present analysis. Further study is needed in order to examine the effects of disease severity and their clinical significance.

A strength of this study is the inclusion of three areas (metropolitan, suburban, and rural areas). Another strength is the use of KYCL subcategories. Hence, our study was able to identify area differences in frailty and factors associated with these differences. However, this study has some limitations. First, urbanization was categorized by using the Japanese national census. Although the generalizability of our results is limited by this categorization, the present findings confirmed previously reported urban/rural differences in frailty [8,9,10,11]. Second, the definition of frailty might have affected our results, although the present range for frailty prevalence was comparable to that in previous studies [35,36]. Future studies should investigate whether the results are similar when another definition, such as that of the Cardiovascular Health Study criteria, is used. Third, we did not measure cognitive function, physical activity, diet, or mental health, among other variables, so the effects of those factors on area differences in frailty are unclear. Forth, in this study, we excluded the participants who had missing data. This may lead to biases, although we used approximately 80% of eligible data in each area. Additionally, the number of participants in each area is different and may potentially affect the statistical power. Finally, this study was cross-sectional, and the results cannot be used to establish causality. Therefore, it is unclear whether a factor with a significantly higher APR in an area is related to incident frailty.

## 5. Conclusions

In conclusion, this study confirmed that the area of residence is associated with frailty in multivariable-adjusted models. This association may be explained by area-specific characteristics and factors associated with frailty. Understanding characteristics of the community leads to enhancing effects of a community-level intervention in preventing frailty.

## Figures and Tables

**Table 1:** Characteristics of men in each area.

Characteristic	Western Metropolitan (*n* = 1204)	Eastern Metropolitan (*n* = 1156)	Suburban District A (*n* = 1004)	Suburban District B (*n* = 438)	Rural (*n* = 702)
***Frailty status***					
Frailty, *n* (%)	259 (21.5)	354 (30.6) *	143 (14.2) *	70 (16.0)	155 (22.1)
***Associated factors***					
Age, mean (SD)	74.2 (5.4)	73.7 (5.3)	73.3 (5.4) *	72.6 (6.1) *	74.7 (6.3) *
Underweight, *n* (%)	45 (3.7)	57 (4.9)	39 (3.9)	18 (4.1)	44 (6.3) *
Overweight, *n* (%)	75 (6.2)	100 (8.7)	66 (6.6)	23 (5.3)	58 (8.3)
Hypertension, *n* (%)	632 (52.5)	649 (56.1)	429 (42.7) *	198 (45.2) *	293 (41.7) *
Heart disease, *n* (%)	274 (22.8)	249 (21.5)	155 (15.4) *	69 (15.8) *	83 (11.8) *
Stroke, *n* (%)	71 (5.9)	85 (7.4)	63 (6.3)	27 (6.2)	35 (5.0)
Diabetes, *n* (%)	217 (18.0)	232 (20.1)	159 (15.8)	73 (16.7)	105 (15.0)
Cancer, *n* (%)	238 (19.8)	191 (16.5)	145 (14.4) *	44 (10.0) *	50 (7.1) *
Bone and joint disease, *n* (%)	210 (17.4)	246 (21.3)	80 (8.0) *	37 (8.4) *	76 (10.8) *
Living alone, *n* (%)	105 (8.7)	176 (15.2) *	78 (7.8)	41 (9.4)	140 (19.9) *
Subjective economic status (poor), *n* (%)	220 (18.3)	319 (27.6) *	237 (23.6) *	134 (30.6) *	136 (19.4)

* *p* < 0.0125 (compared to the western metropolitan area).

**Table 2:** Characteristics of women in each area.

Characteristic	Western Metropolitan (*n* = 1232)	Eastern Metropolitan (*n* = 1193)	Suburban District A (*n* = 946)	Suburban District B (*n* = 433)	Rural (*n* = 874)
***Frailty status***					
Frailty, *n* (%)	192 (15.6)	255 (21.4) *	109 (11.5) *	69 (15.9)	173 (19.8) *
***Associated factors***					
Age, mean (SD)	73.8 (5.5)	73.5 (5.3)	72.4 (5.7) *	73.1 (6.9)	74.9 (6.2) *
Underweight, *n* (%)	173 (14.0)	98 (8.2) *	104 (11.0) *	47 (10.9)	73 (8.4) *
Overweight, *n* (%)	61 (5.0)	121 (10.1) *	53 (5.6)	31 (7.2)	87 (10.0) *
Hypertension, *n* (%)	533 (43.3)	610 (51.1) *	350 (37.0) *	208 (48.0)	380 (43.5)
Heart disease, *n* (%)	150 (12.2)	137 (11.5)	60 (6.3) *	31 (7.2) *	68 (7.8) *
Stroke, *n* (%)	44 (3.6)	44 (3.7)	26 (2.7)	15 (3.5)	20 (2.3)
Diabetes, *n* (%)	92 (7.5)	128 (10.7) *	76 (8.0)	56 (12.9) *	87 (10.0)
Cancer, *n* (%)	137 (11.1)	154 (12.9)	105 (11.1)	32 (7.4)	49 (5.6) *
Bone and joint disease, *n* (%)	408 (33.1)	457 (38.3) *	181 (19.1) *	67 (15.5) *	195 (22.3) *
Living alone, *n* (%)	232 (18.8)	229 (19.2)	126 (13.3) *	49 (11.3) *	260 (29.7) *
Subjective economic status (poor), *n* (%)	204 (16.6)	286 (24.0) *	199 (21.0) *	123 (28.4) *	161 (18.4)

* *p* < 0.0125 (compared to the western metropolitan area).

**Table 3:** Scores for Kaigo-Yobo Checklist (KYCL) subcategories in each area.

Variable	Western Metropolitan	Eastern Metropolitan	Suburban District A	Suburban District B	Rural
	Mean (SD)	Mean (SD)	Mean (SD)	Mean (SD)	Mean (SD)
***Men***					
Fall	0.67 (0.93)	0.79 (1.00)	0.48 (0.83) *	0.64 (0.95)	0.71 (1.00)
Nutritional status	0.46 (0.70)	0.63 (0.83) *	0.45 (0.69)	0.44 (0.74)	0.56 (0.79) *
Social activities	1.17 (1.12)	1.31 (1.25)	0.77 (1.02) *	0.69 (1.08) *	0.92 (1.22) *
***Women***					
Fall	0.66 (0.95)	0.80 (1.00)	0.54 (0.88)	0.73 (1.04)	0.77 (0.98)
Nutritional status	0.41 (0.66)	0.51 (0.74) *	0.40 (0.69)	0.49 (0.78)	0.49 (0.70) *
Social activities	0.75 (0.98)	0.92 (1.10) *	0.51 (0.86) *	0.57 (0.99) *	0.85 (1.16)

* *p* < 0.0125 (compared to the western metropolitan area). The lower scores indicate a better function. An analysis of covariance (ANCOVA) adjusted for age, underweight, overweight, medical histories (hypertension, heart disease, stroke, diabetes mellitus, cancer, and bone and joint diseases), living arrangement (living alone), and subjective economic status was used.

**Table 4:** The association between living area and frailty.

Residential Area	Age-Adjusted PR (95% CI)	Multivariable-Adjusted PR (95% CI)
***Men***		
Western metropolitan	1.00 (reference)	1.00 (reference)
Eastern metropolitan	**1.45 (1.26–1.66)**	**1.27 (1.11–1.46)**
Suburban district A	**0.68 (0.57–0.82)**	**0.73 (0.61–0.88)**
Suburban district B	**0.78 (0.62–0.99)**	**0.79 (0.63–1.00)**
Rural	1.01 (0.84–1.20)	1.09 (0.92–1.30)
***Women***		
Western metropolitan	1.00 (reference)	1.00 (reference)
Eastern metropolitan	**1.40 (1.19–1.66)**	**1.31 (1.12–1.55)**
Suburban district A	**0.79 (0.64–0.98)**	0.86 (0.69–1.06)
Suburban district B	1.03 (0.80–1.31)	1.07 (0.84–1.36)
Rural	1.17 (0.97–1.41)	**1.38 (1.14–1.65)**

PR: prevalence ratio. CI: confidence interval. Multivariable-adjusted model includes age, underweight, overweight, medical histories (hypertension, heart disease, stroke, diabetes, cancer, and bone and joint diseases), living arrangement (living alone), and subjective economic status as covariates. Bold numbers indicate *p* < 0.05.

## Supplementary Materials

The following are available online at https://www.mdpi.com/1660-4601/16/20/3974/s1: Figure S1: Flow chart of date collection, Table S1: Kaigo-Yobo Checklist, Table S2: The association between each variable and frailty in each area.
