# Supplementary material for: Differences in the Prevalence of and Factors Associated with Frailty in Five Japanese Residential Areas
Source: Int J Environ Res Public Health. 2019 Oct 18;16(20):3974. doi: 10.3390/ijerph16203974 (PMC6843904; doi:10.3390/ijerph16203974)
Supplement: Supplementary file 1 [file ijerph-16-03974-s001.zip › SM/Supplementary Table S2_IJERPH.docx]

| Supplementary Table 2. The association between each variable and frailty in each area | | | | | | | | | | | | | | | | | | | | | | | | | | | | | | | | | | | | | | | | | | | |
| --- | --- | --- | --- | --- | --- | --- | --- | --- | --- | --- | --- | --- | --- | --- | --- | --- | --- | --- | --- | --- | --- | --- | --- | --- | --- | --- | --- | --- | --- | --- | --- | --- | --- | --- | --- | --- | --- | --- | --- | --- | --- | --- | --- |
|  | |  | | | | All | | | | | | | | | | | | | | | | | |  |  | | Western metropolitan | | | | | | | | | | | | | | | | |
|  | |  | | | | APR (95% CI) | | | | | | | | | | | | | | | | | |  |  | | APR (95% CI) | | | | | | | | | | | | | | | | |
| ***Men*** | | | | | |  | | | |  | |  | | | |  | |  | | | | | |  |  | |  | | | |  | |  | | | |  | |  | | | |  |
|  | | Age (1 year) | | | | **1.03** (**1.02–1.04**) | | | | | | | | | | | | | | | | | | |  | | **1.04** (**1.02–1.06**  ) | | | | | | | | | | | | | | | | |
|  | | Underweight | | | | **1.71** (**1.42**–**2.05**) | | | | | | | | | | | | | | | | | | |  | | 1.34 (0.86–2.09) | | | | | | | | | | | | | | | | |
|  | | Overweight | | | | **1.36** (**1.16**–**1.61**) | | | | | | | | | | | | | | | | | | |  | | **1.41** (**1.00**–**1.98**) | | | | | | | | | | | | | | | | |
|  | | Hypertension | | | | **1.20** (**1.07**–**1.34**) | | | | | | | | | | | | | | | | | | |  | | 1.14 (0.92–1.43) | | | | | | | | | | | | | | | | |
|  | | Heart disease | | | | **1.21** (**1.07**–**1.37**) | | | | | | | | | | | | | | | | | | |  | | **1.47** (**1.18**–**1.84**) | | | | | | | | | | | | | | | | |
|  | | Stroke | | | | **1.45** (**1.23**–**1.72**) | | | | | | | | | | | | | | | | | | |  | | 1.12 (0.79–1.59) | | | | | | | | | | | | | | | | |
|  | | Diabetes | | | | **1.27** (**1.12**–**1.43**) | | | | | | | | | | | | | | | | | | |  | | **1.34** (**1.05**–**1.70**) | | | | | | | | | | | | | | | | |
|  | | Cancer | | | | **1.37** (**1.20**–**1.56**) | | | | | | | | | | | | | | | | | | |  | | **1.40** (**1.10**–**1.78**) | | | | | | | | | | | | | | | | |
|  | | Bone and joint disease | | | | **1.46** (**1.29**–**1.65**) | | | | | | | | | | | | | | | | | | |  | | **1.49** (**1.19**–**1.87**) | | | | | | | | | | | | | | | | |
|  | | Living alone | | | | **1.30** (**1.13**–**1.49**) | | | | | | | | | | | | | | | | | | |  | | **1.70** (**1.29**–**2.23**) | | | | | | | | | | | | | | | | |
|  | | Subjective economic  status (poor) | | | | **1.84** (**1.65**–**2.05**) | | | | | | | | | | | | | | | | | | |  | | **1.95** (**1.57**–**2.43**) | | | | | | | | | | | | | | | | |
| ***Women*** | | | | | |  | | | | | | | | | | | | | | | | | | |  | |  | | | | | | | | | | | | | | | | |
|  | | Age (1 year) | | | | **1.06** (**1.04–1.07**) | | | | | | | | | | | | | | | | | | |  | | **1.08** (**1.05–1.11**) | | | | | | | | | | | | | | | | |
|  | | Underweight | | | | **1.37** (**1.15**–**1.63**) | | | | | | | | | | | | | | | | | | |  | | 1.23 (0.89–1.71) | | | | | | | | | | | | | | | | |
|  | | Overweight | | | | 1.09 (0.88–1.35) | | | | | | | | | | | | | | | | | | |  | | 0.90 (0.52–1.56) | | | | | | | | | | | | | | | | |
|  | | Hypertension | | | | 0.97(0.85–1.11) | | | | | | | | | | | | | | | | | | |  | | 1.00(0.77–1.31) | | | | | | | | | | | | | | | | |
|  | | Heart disease | | | | **1.46**(**1.25**–**1.71**) | | | | | | | | | | | | | | | | | | |  | | **1.44**(**1.08**–**1.93**) | | | | | | | | | | | | | | | | |
|  | | Stroke | | | | **1.70**(**1.35**–**2.13**) | | | | | | | | | | | | | | | | | | |  | | 1.30(0.85–1.99) | | | | | | | | | | | | | | | | |
|  | | Diabetes | | | | **1.35**(**1.13**–**1.61**) | | | | | | | | | | | | | | | | | | |  | | 1.24(0.83–1.84) | | | | | | | | | | | | | | | | |
|  | | Cancer | | | | **1.40**(**1.18**–**1.66**) | | | | | | | | | | | | | | | | | | |  | | **1.40**(**1.02**–**1.93**) | | | | | | | | | | | | | | | | |
|  | | Bone and joint disease | | | | **1.54**(**1.36**–**1.75**) | | | | | | | | | | | | | | | | | | |  | | **1.67**(**1.31**–**2.15**) | | | | | | | | | | | | | | | | |
|  | | Living alone | | | | **0.84**(**0.72**–**0.98**) | | | | | | | | | | | | | | | | | | |  | | 0.86(0.62–1.18) | | | | | | | | | | | | | | | | |
|  | | Subjective economic  status (poor) | | | | **1.95**(**1.72**–**2.21**) | | | | | | | | | | | | | | | | | | |  | | **2.38**(**1.84**–**3.08**) | | | | | | | | | | | | | | | | |
| Supplementary Table 1. (cont.) | | | | | | | | | | | | | | | | | | | | | | | | | | | | | | | | | | | | | | | | |  |  |  |
|  |  | | Eastern metropolitan | | | | | | | | | | | | | | | | |  | Suburban district A | | | | | | | | | | | | | | | | | | | |  |  |  |
|  |  | | APR (95% CI) | | | | | | | | | | | | | | | | |  | APR (95% CI) | | | | | | | | | | | | | | | | | | | |  |  |  |
| ***Men*** | | |  | |  | | |  | | |  | | |  | | |  | | |  |  | | | | |  | | |  | | |  | | |  | | |  | | |  |  |  |
|  | Age (1 year) | | 1.02(1.00**–**1.03) | | | | | | | | | | | | | | | | |  | 1.03(1.00**–**1.05) | | | | | | | | | | | | | | | | | | | |  |  |  |
|  | Underweight | | **1.69**(**1.30**–**2.20**) | | | | | | | | | | | | | | | | |  | **1.92**(**1.09**–**3.38**) | | | | | | | | | | | | | | | | | | | |  |  |  |
|  | Overweight | | **1.46**(**1.16**–**1.83**) | | | | | | | | | | | | | | | | |  | 1.22(0.70–2.13) | | | | | | | | | | | | | | | | | | | |  |  |  |
|  | Hypertension | | 1.12(0.93–1.34) | | | | | | | | | | | | | | | | |  | 1.30(0.96–1.77) | | | | | | | | | | | | | | | | | | | |  |  |  |
|  | Heart disease | | 1.05(0.87–1.28) | | | | | | | | | | | | | | | | |  | 1.21(0.82–1.78) | | | | | | | | | | | | | | | | | | | |  |  |  |
|  | Stroke | | **1.48**(**1.16**–**1.87**) | | | | | | | | | | | | | | | | |  | **2.30**(**1.51**–**3.49**) | | | | | | | | | | | | | | | | | | | |  |  |  |
|  | Diabetes | | **1.26**(**1.04**–**1.51**) | | | | | | | | | | | | | | | | |  | **1.56**(**1.11**–**2.18**) | | | | | | | | | | | | | | | | | | | |  |  |  |
|  | Cancer | | 1.21(0.99–1.49) | | | | | | | | | | | | | | | | |  | **1.63**(**1.14**–**2.33**) | | | | | | | | | | | | | | | | | | | |  |  |  |
|  | Bone and joint disease | | **1.23**(**1.02**–**1.48**) | | | | | | | | | | | | | | | | |  | **2.23**(**1.52**–**3.28**) | | | | | | | | | | | | | | | | | | | |  |  |  |
|  | Living alone | | **1.31**(**1.07**–**1.61**) | | | | | | | | | | | | | | | | |  | 1.26(0.77–2.05) | | | | | | | | | | | | | | | | | | | |  |  |  |
|  | Subjective economic  status (poor) | | **1.79**(**1.52**–**2.12**) | | | | | | | | | | | | | | | | |  | **1.69**(**1.25**–**2.30**) | | | | | | | | | | | | | | | | | | | |  |  |  |
| ***Women*** | | |  | |  | | |  | | |  | | |  | | |  | | |  |  | | | | |  | | |  | | |  | | |  | | |  | | |  |  |  |
|  | Age (1 year) | | **1.05**(**1.02–1.07**) | | | | | | | | | | | | | | | | |  | **1.07**(**1.05–1.10**) | | | | | | | | | | | | | | | | | | | |  |  |  |
|  | Underweight | | 1.20(0.83–1.72) | | | | | | | | | | | | | | | | |  | **1.60**(**1.06**–**2.41**) | | | | | | | | | | | | | | | | | | | |  |  |  |
|  | Overweight | | 1.30(0.94–1.79) | | | | | | | | | | | | | | | | |  | 1.03(0.53–1.98) | | | | | | | | | | | | | | | | | | | |  |  |  |
|  | Hypertension | | 1.07(0.85–1.35) | | | | | | | | | | | | | | | | |  | 0.76(0.54–1.08) | | | | | | | | | | | | | | | | | | | |  |  |  |
|  | Heart disease | | 1.26(0.96–1.67) | | | | | | | | | | | | | | | | |  | **1.82**(**1.20**–**2.75**) | | | | | | | | | | | | | | | | | | | |  |  |  |
|  | Stroke | | 1.22(0.75–1.98) | | | | | | | | | | | | | | | | |  | **2.36**(**1.31**–**4.26**) | | | | | | | | | | | | | | | | | | | |  |  |  |
|  | Diabetes | | 1.04(0.75–1.43) | | | | | | | | | | | | | | | | |  | **1.74**(**1.04**–**2.89**) | | | | | | | | | | | | | | | | | | | |  |  |  |
|  | Cancer | | 1.30(0.98–1.72) | | | | | | | | | | | | | | | | |  | 1.58(0.98–2.57) | | | | | | | | | | | | | | | | | | | |  |  |  |
|  | Bone and joint disease | | 1.16(0.93–1.44) | | | | | | | | | | | | | | | | |  | **2.71**(**1.93**–**3.81**) | | | | | | | | | | | | | | | | | | | |  |  |  |
|  | Living alone | | 0.79(0.59–1.05) | | | | | | | | | | | | | | | | |  | 0.72(0.43–1.20) | | | | | | | | | | | | | | | | | | | |  |  |  |
|  | Subjective economic  status (poor) | | **2.04**(**1.65**–**2.53**) | | | | | | | | | | | | | | | | |  | 1.16(0.79–1.70) | | | | | | | | | | | | | | | | | | | |  |  |  |
| Supplementary Table 1. (cont.) | | | | | | | | | | | | | | | | | | | | | | | | | | | | | | | | | | | | | | | | | |  |  |
|  |  | | | Suburban district B | | | | | | | | | | | | | | | | | |  | Rural | | | | | | | | | | | | | | | | | | |  |  |
|  |  | | | APR (95% CI) | | | | | | | | | | | | | | | | | |  | APR (95% CI) | | | | | | | | | | | | | | | | | | |  |  |
| ***Men*** | | | |  | | |  | |  | | | |  | |  | | | |  | | |  |  | | | | |  | |  | | | |  | |  | | | |  | |  |  |
|  | Age (1 year) | | | 1.03(0.99**–**1.07) | | | | | | | | | | | | | | | | | |  | **1.03**(**1.01–1.05**) | | | | | | | | | | | | | | | | | | |  |  |
|  | Underweight | | | **2.06**(**1.05**–**4.02**) | | | | | | | | | | | | | | | | | |  | **1.74**(**1.15**–**2.64**) | | | | | | | | | | | | | | | | | | |  |  |
|  | Overweight | | | 1.54(0.72–3.30) | | | | | | | | | | | | | | | | | |  | 1.35(0.90–2.05) | | | | | | | | | | | | | | | | | | |  |  |
|  | Hypertension | | | 1.50(0.96–2.34**)** | | | | | | | | | | | | | | | | | |  | 1.20(0.91–1.59) | | | | | | | | | | | | | | | | | | |  |  |
|  | Heart disease | | | 1.29(0.79–2.11) | | | | | | | | | | | | | | | | | |  | 1.36(0.96–1.94) | | | | | | | | | | | | | | | | | | |  |  |
|  | Stroke | | | 1.74(0.91–3.34) | | | | | | | | | | | | | | | | | |  | 1.36(0.82–2.28) | | | | | | | | | | | | | | | | | | |  |  |
|  | Diabetes | | | 1.15(0.70–1.89) | | | | | | | | | | | | | | | | | |  | 1.06(0.74–1.53) | | | | | | | | | | | | | | | | | | |  |  |
|  | Cancer | | | 1.60(0.97–2.66) | | | | | | | | | | | | | | | | | |  | **1.73**(**1.22**–**2.45**) | | | | | | | | | | | | | | | | | | |  |  |
|  | Bone and joint disease | | | **2.79**(**1.73**–**4.47**) | | | | | | | | | | | | | | | | | |  | **1.40**(**1.00**–**1.95**) | | | | | | | | | | | | | | | | | | |  |  |
|  | Living alone | | | 1.26(0.66–2.43) | | | | | | | | | | | | | | | | | |  | 0.91(0.66–1.27) | | | | | | | | | | | | | | | | | | |  |  |
|  | Subjective economic  status (poor) | | | **1.63**(**1.05**–**2.52**) | | | | | | | | | | | | | | | | | |  | **2.28**(**1.73**–**3.01**) | | | | | | | | | | | | | | | | | | |  |  |
| ***Women*** | | | |  | | |  | |  | | | |  | |  | | | |  | | |  |  | | | | |  | |  | | | |  | |  | | | |  | |  |  |
|  | Age (1 year) | | | **1.07**(**1.04–1.11**) | | | | | | | | | | | | | | | | | |  | **1.03**(**1.01–1.05**) | | | | | | | | | | | | | | | | | | |  |  |
|  | Underweight | | | **2.25**(**1.37**–**3.71**) | | | | | | | | | | | | | | | | | |  | 1.36(0.92–2.01) | | | | | | | | | | | | | | | | | | |  |  |
|  | Overweight | | | 0.68(0.25–1.83) | | | | | | | | | | | | | | | | | |  | 1.02(0.68–1.53) | | | | | | | | | | | | | | | | | | |  |  |
|  | Hypertension | | | 1.02(0.67–1.56**)** | | | | | | | | | | | | | | | | | |  | 0.88(0.67–1.15) | | | | | | | | | | | | | | | | | | |  |  |
|  | Heart disease | | | 1.62(0.90–2.90) | | | | | | | | | | | | | | | | | |  | **1.60**(**1.13**–**2.29**) | | | | | | | | | | | | | | | | | | |  |  |
|  | Stroke | | | **2.78**(**1.23**–**6.29**) | | | | | | | | | | | | | | | | | |  | **2.60**(**1.72**–**3.93**) | | | | | | | | | | | | | | | | | | |  |  |
|  | Diabetes | | | **2.21**(**1.34**–**3.65**) | | | | | | | | | | | | | | | | | |  | **1.58**(**1.10**–**2.28**) | | | | | | | | | | | | | | | | | | |  |  |
|  | Cancer | | | **2.29**(**1.16**–**4.53**) | | | | | | | | | | | | | | | | | |  | 1.27(0.80–2.01) | | | | | | | | | | | | | | | | | | |  |  |
|  | Bone and joint disease | | | **2.44**(**1.53**–**3.89**) | | | | | | | | | | | | | | | | | |  | **1.39**(**1.05**–**1.86**) | | | | | | | | | | | | | | | | | | |  |  |
|  | Living alone | | | 1.21(0.67–2.20) | | | | | | | | | | | | | | | | | |  | 0.88(0.67–1.17) | | | | | | | | | | | | | | | | | | |  |  |
|  | Subjective economic  status (poor) | | | 1.33(0.87–2.05) | | | | | | | | | | | | | | | | | |  | **2.10**(**1.59**–**2.79**) | | | | | | | | | | | | | | | | | | |  |  |
| APR: adjusted prevalence ratio. CI: confidence interval. Bold numbers indicate *p* < 0.05. | | | | | | | | | | | | | | | | | | | | | | | | | | | | | | | | | | | | | | | | | |  |  |
