# Supplementary material for: Differences in the Prevalence of and Factors Associated with Frailty in Five Japanese Residential Areas
Source: Int J Environ Res Public Health. 2019 Oct 18;16(20):3974. doi: 10.3390/ijerph16203974 (PMC6843904; doi:10.3390/ijerph16203974)
Supplement: Supplementary file 1 [file ijerph-16-03974-s001.zip › SM/Table S1.docx]

Supplementary Table S1.

Kaigo-Yobo Checklist

Falling

-Have you experienced a fall in the past year?

-Can you walk for 1 km?

-Can you see things clearly? (with glasses if necessary)

-Do you often slip or stumble at home?

-Do you refrain from going out because of fear of falling?

-Have you been hospitalized in the past year?

Nutritional status

-Do you have appetite these days?

-Do you have any difficulty chewing? (even with a denture)

-Have you lost 3 kg or more in the past 6 months?

-Do you think you have lost muscle or fat in the past 6 months?

Social activities

-Do you usually stay at home all day long?

-How often do you usually go out? (more than once per 2–3 days or less than once a week)

-Do you have any hobby?

-Do you have neighbors who you can talk closely with?

-Besides your neighbors, do you have close friends, families, or relatives who you visit?

Ref.

Shinkai, S.; Yoshida, H.; Taniguchi, Y.; Murayama, H.; Nishi, M.; Amano, H.; Nofuji, Y.; Seino, S.; Fujiwara, Y. Public health approach to preventing frailty in the community and its effect on healthy aging in Japan. Geriatr. Gerontol. Int. 2016, 16, 87–97.

Kojima, G.; Taniguchi, Y.; Kitamura, A.; Shinkai, S. Are the Kihon Checklist and the Kaigo-Yobo Checklist Compatible With the Frailty Index? J. Am. Med. Dir. Assoc. 2018, 19, 797-800.e2.
