# Supplementary figures and images for: Differences in the Prevalence of and Factors Associated with Frailty in Five Japanese Residential Areas
Source: Int J Environ Res Public Health. 2019 Oct 18;16(20):3974. doi: 10.3390/ijerph16203974 (PMC6843904; doi:10.3390/ijerph16203974)

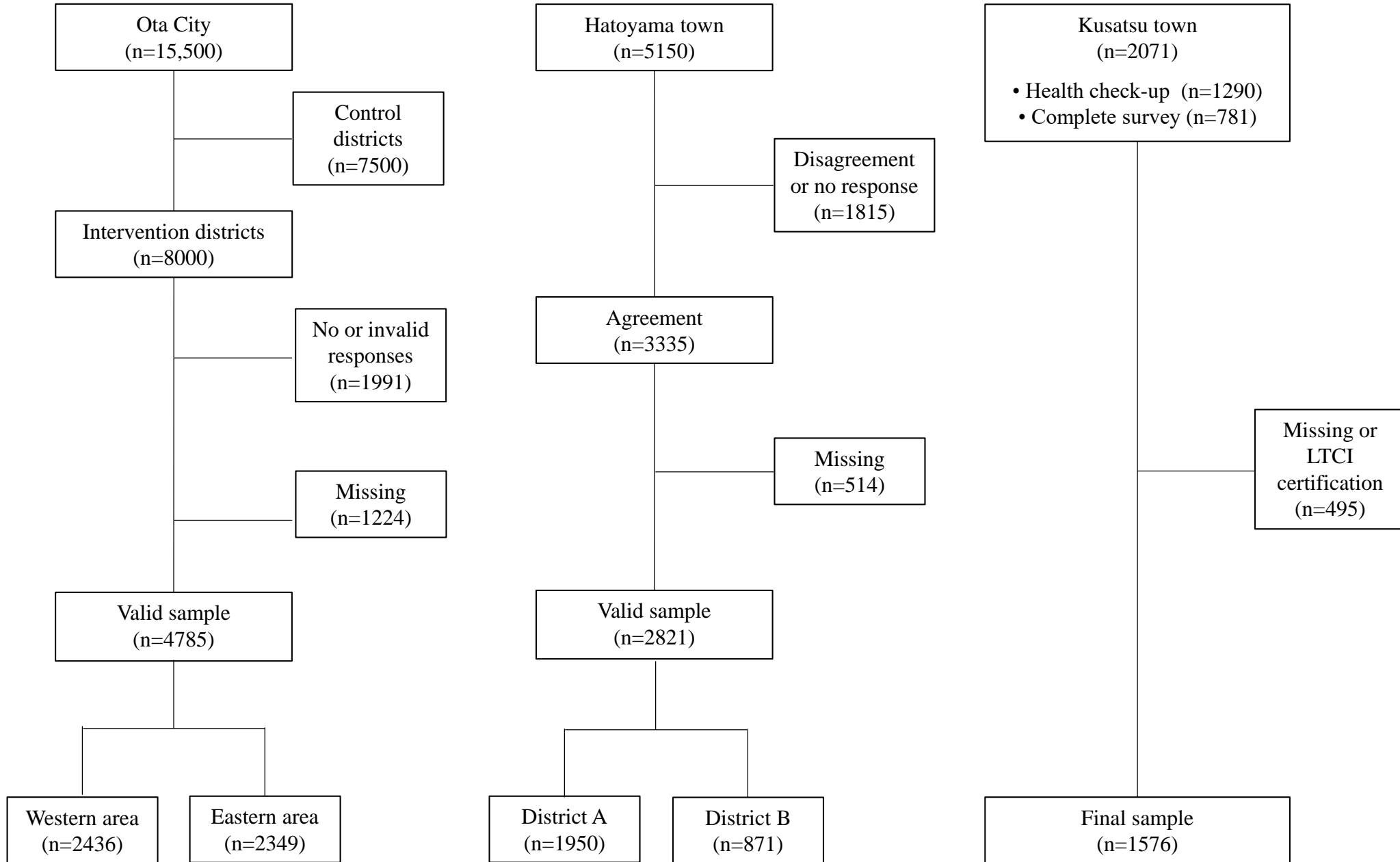

Supplementary Figure 1. Flow chart of data collection.  
LTCI: long-term care insurance

Supplement: Supplementary file 1 [file ijerph-16-03974-s001.zip › SM/Figure S1.pdf]
